# Design and implementation of an innovative single-phase direct AC-AC bipolar voltage buck converter with enhanced control topology

**DOI:** 10.1038/s41598-024-66626-5

**Published:** 2024-07-10

**Authors:** Naveed Ashraf, Ghulam Abbas, Zohaib Mushtaq, Ateeq Ur Rehman, Khmaies Ouahada, Habib Hamam

**Affiliations:** 1 Department of Electrical Engineering, The University of Lahore, Lahore, 54000 Pakistan; 2 Department of Electrical Engineering, College of Engineering and Technology, University of Sargodha, Sargodha, 40100 Pakistan; 3 School of Computing, Gachon University, Seongnam, 13120 Republic of Korea; 4 Department of Electrical and Electronic Engineering Science, School of Electrical Engineering, University of Johannesburg, Johannesburg, 2006 South Africa; 5 Faculty of Engineering, Uni de Moncton, Moncton, NB E1A3E9 Canada; 6 Hodmas University College, Taleh Area, Mogadishu, Somalia; 7 Bridges for Academic Excellence, Centre Ville, 1002 Tunis, Tunisia

**Keywords:** AC–AC converter, DC rail capacitor, Voltage and frequency controller, Voltage buck operation, Voltage and current ripples, Bipolar output voltage, Pulse width modulation, Non-inverted and inverted outputs, Energy science and technology, Engineering

## Abstract

Direct AC–AC converters are strong candidates in the power converting system to regulate grid voltage against the perturbation in the line voltage and to acquire frequency regulation at discrete step levels in variable speed drivers for industrial systems. All such applications require the inverted and non-inverted form of the input voltage across the output with voltage-regulating capabilities. The required value of the output frequency is gained with the proper arrangement of the number of positive and negative pulses of the input voltage across the output terminals. The period of each such pulse for low-frequency operation is almost the same as the half period of the input grid or utility voltage. These output pulses are generated by converting the positive and negative input half cycles in noninverting and inverting forms as per requirement. There is no control complication to generate control signals used to adjust the load frequency as the operating period of the switching devices is normally greater than the period of the source voltage. However, high-frequency pulse width modulated (PWM) control signals are used to regulate the output voltage. The size of the inductor and capacitor is inversely related to the value of the switching frequency. Similarly, the ripple contents of voltage and currents in these filtering components are also inversely linked with PWM frequency. These constraints motivate the circuit designer to select high PWM frequency. However, the alignment of the high-frequency control input with the variation in the input source voltage is a big challenge for a design engineer as the switching period of a high-frequency signal normally lies in the microsecond. It is also required to operate some high-frequency devices for various half cycles of the source voltage, creating control complications as the polarities of the half cycles are continuously changing. This requires at least the generation of two high-frequency signals for different intervals. The interruption of the filtering inductor current is a big source of high voltage surges in circuits where the high-frequency transistors operate in a complementary way. This may be due to internal defects in the switching transistors or some unnecessary inherent delay in their control signals. In this research work, a simplified AC–AC converter is developed that does not need alignment of high-frequency control with the polarity of the source voltage. With this approach, high-frequency signals can be generated with the help of any analog or digital control system. By applying this technique, only one high-frequency control signal is generated and applied in AC circuits, as in a DC converter, without applying a highly sensitive polarity sensing circuit. So, controlling complications is drastically simplified. The circuit and configuration always avoid the current interruption problem of filtering the inductor. The proposed control and circuit topology are tested both in computer-based simulation and practically developed circuits. The results obtained from these platforms endorse the effectiveness and validation of the proposed work.

## Introduction

Direct conversion of AC power for single-phase applications includes the regulation of voltage and frequency. Enormous applications require voltage control to drive their load successfully and smoothly. For example, the speed control mechanism of small power-rating loads such as fans, blowers, and pump motors needs the control of RMS voltage. Such a requirement only needs unipolar voltage control. However, some applications require the control of output RMS voltage in a bipolar manner. Applications requiring voltage regulation with dual polarity include grid voltage correction in the event of voltage sag and swell. These problems are triggered by the system’s faults, such as a ground fault in the three-phase distribution network. This event results in the decrease of line voltage in the faulty line and an increase in voltage in the remaining non-faulty lines. These problems are very sensitive to critical loads, so voltage correction is mandatory. The voltage compensation of such a problem is obtained by adding and subtracting the voltage in the line for voltage sag and swell problems, respectively. With the ability to switch between noninverting and inverting modes, power-supplying converters may effortlessly guarantee such injected voltage characteristics. Such converters can also be employed to arrange the number of positive and negative half cycles across the load to govern frequency at discrete step levels. Application of such outputs includes the speed control of induction motors employed in steel rolling and grinding mills. All of this points to how critical it is to develop a bipolar voltage gain single-phase converter. A simple circuit and control strategy is a key requirement in this application area.

AC–AC power converters can be categorized into two groups. The first group utilizes the indirect power conversion approach, which is AC–DC–AC conversion^1–5^. A rectifier circuit is associated at the first stage of this converter for the conversion of AC power to DC. Its output consists of DC components and even harmonics. The most challenging harmonic is the second-order harmonic component, which degrades the performance of the second-stage inverter used to convert DC voltage to AC form. The intermediate power conversion stage of this group is DC, where power is stored in a DC-rail capacitor. This acts as a coupling element between the input and output. It also serves as a filtering element, but its size is bulky. Short lifetime and low reliability are the big challenges of this capacitor. The voltage regulation in the output AC voltage may be controlled with the help of an intermediate DC–DC converter. The second harmonic component is a big source of high conversion losses in the DC-DC power conversion process^5^. The main application area of the indirect AC–DC–AC converters is the variable-speed industrial drive to control the speed of the rotating loads. It is inefficient to use such converters as voltage controllers. Some of the deficiencies of the indirect AC–DC–AC are tackled by applying the multilevel approach, as discussed in Refs^6,7^. The converter in Ref^6^ is a five-level, and its operation is based on the use of a high-frequency transformer. In Ref^7^, three modules with two DC link capacitors are employed, and the problem of floating capacitors is tackled. All indirect AC-to-AC converters suffer from circuit complexity, an increase in overall volume, high cost, and low conversion efficiency. Such topologies are not attractive in applications that need voltage control and step variation in frequencies.

The second category of AC–AC converters utilizes the direct power conversion approach, and its realization is free from the use of bulky and unreliable DC rail capacitors. Here, input AC power is directly transformed into regulated output AC power. This power conversion process may utilize the z-source components or voltage buck-boost approach for the regulation of the output voltage. There are two subclasses of this approach depending on the polarity of the output voltage. In a unipolar voltage gain converter, as reported in Ref^8,9^, the phase shift between the input and output voltage is normally zero. Generally, these converters are used for voltage control applications. This task may be completed with the firing delay control of the thyristors. In this process, the frequency of the generated harmonics is low^8^, increasing the filtering elements’ size. It can be lowered by increasing the switching frequency with pulse width control (PWM). The unipolar voltage gain characteristics of the output voltage restrict its application in some limited applications. In bipolar voltage gain converters, the output voltage can be in-phase or output-phase, depending on the input voltage. The output of such characteristics can be achieved with the control signals. Z-source is a network of impedance inserted between the input source and circuit of the solid electronic devices^10–14^. This structure facilitates the regulation of output voltage and phase angle, meaning bipolar output can be produced. The main concern faced by the design engineer in these converters is the presence of shoot-through intervals. There is the conduction of high-current surges during the said intervals. This problem increases the current rating of the operating devices and overall cost^15–17^.

The traditional voltage buck topology employed in DC–DC converters may also be effectively employed in direct AC–AC converters^18^. In this circuit, unidirectional switching devices of the DC–DC converter are replaced with bidirectional current conduction devices. These devices help to process both polarities of the input voltage. The output voltage of this circuit is always in phase with the input voltage and is a non-inverted output concerning the input. This developed circuit is tested to solve the problem of voltage sag, which results in the power systems due to the single-line to-ground faults. During this fault event, the instantaneous voltage of the line where the fault occurs decreases concerning their set values while the voltage of the other lines increases above their rated values. The increase and decrease in the instantaneous voltage in power quality terminology are called voltage sag and swell, respectively. In most cases, the voltage depth for the voltage sag problem is less than 50% of the rated value. Normally, the voltage depth or change level of voltage swell is less than that of voltage sag. A voltage buck circuit is a strong candidate to use as a dynamic voltage restorer. In voltage buck operation, the change in the output voltage can be set from the minimum value of the input voltage to its maximum value through the PWM control. The voltage gains of the voltage buck circuit presented in Ref^18^ are unipolar, which can only resolve voltage sag or swell but not both. The direct AC–AC converters having voltage no-inverted and inverted features are reported in Ref^19,20^. Voltage buck circuits of Refs^19,20^ use eight and six switching transistors as fully controlled devices. The circuits have bipolar output voltage control; it can also be effectively used to govern the output frequency at a discrete level, as given in Refs^21–26^. In step-down variable frequency operation, proper voltage regulation is mandatory to maintain the flux of the machine constant; otherwise, the core may saturate. Reduced component voltage buck-boost topologies are introduced in Refs^27,28^. In Ref^27^, dual IGBT modules are employed, and their operating periods control the desired output voltage. The voltage gain of these two converters is unipolar. In the present form, these converters can only be operated in a non-inverted form. Therefore, they can only tackle the voltage sag or swell problem, not both. They cannot also be employed as frequency controllers. A common ground facility is available in the converter mentioned in Ref^28^, but two inductors are used. This converter can only be operated in noninverting buck-boost form. Although this converter cannot suffer the shoot-through problem but it is not independent of the current commutation problem. For example, in this circuit, four IGBTs have antiparallel diodes operating in two pairs in each cycle of the source voltage. Here, IGBT *S*_1_ and *S*_4_ operate in a complementary way. The same is also true for IGBTs *S*_2_ and *S*_3_. For the positive half cycle of the input voltage, IGBT *S*_1_ is conducted, and filtering inductor *L*_1_ gets energized from source voltage and capacitor *C*_1_ with the forward biasing of diode *D*_2_. In this interval, IGBT *S*_4_ remains off. Now, the stored energy of the inductor is to be transferred to load by turning on and off IGBTs *S*_4_ and *S*_1,_ respectively. However, due to defects or manufacturing constraints in the IGBTs *S*_1_ and *S*_4_, there is a chance that the IGBT *S*_1_ turns off before turning on the IGBT *S*_4_. This action may cause the current interruption of inductor L1 and create high inductive voltage surges. The same issue may happen during the negative half cycle of the input voltage due to the complementary operation of IGBTs *S*_2_ and *S*_3_. The induction of high voltage inductive surges can cause damage or failure to the semiconductor devices, and there is a need for a protection arrangement to deal with such issues. The converter presented in Ref^29^ can perform voltage boost operation with high voltage by cascading a number of units. Two bidirectional current-conducting switching modules, an inductor and a capacitor, are used in each unit. The voltage stresses across the switching units can be lowered by increasing the number of cascading modules, but with this approach, the number of components is increased. Gate control techniques, especially high-frequency control inputs, which are responsible for the regulation of the voltage gain or output has to be synchronized with the polarity variation of the source voltage. The voltage gain control is unipolar, which means these converters^27–29^ can only perform noninverting operations, and they are not attracted in applications where bipolar voltage gain control is required.

In the control strategy for the proposed single-phase bipolar voltage buck converter, the use of Proportional-Integral (PI) and Fuzzy-Logic (FL) controllers can significantly enhance the performance and adaptability of the system under varying conditions. According to Chakroun and Hamam^30^, employing PI and FL controllers in energy conversion systems has proven to offer high efficiency and dynamic response adaptability, especially in scenarios involving energy recovery from used batteries^31^. The fuzzy-logic controller, in particular, can adapt to the nonlinear characteristics of buck converters under different operating conditions, providing an innovative approach to controlling the output voltage and frequency of our proposed converter.

Furthermore, the integration of machine learning techniques for monitoring and predictive maintenance of power converters in smart grid applications, as explored by Sadiq et al., aligns with our proposed design’s emphasis on reliability and efficiency^31^. The application of machine learning algorithms for real-time monitoring can significantly enhance the detection of potential issues and optimize maintenance strategies. This is particularly relevant in the context of our converter, where the health of passive components like inductors and capacitors directly influences performance. By leveraging a machine learning-based monitoring system, the proposed converter design could predict component degradation and facilitate timely interventions, thus ensuring stable operation and extending the lifespan of the system in smart grid environments. References^30,31^ underscore the importance of advanced control strategies and the potential benefits of incorporating machine learning for monitoring and predictive maintenance in the development of efficient and reliable power converters. By integrating these methodologies, the proposed single-phase bipolar voltage buck converter not only addresses the immediate need for voltage and frequency regulation in industrial applications but also sets the foundation for future advancements in converter technology and smart grid integration.

In reported voltage buck circuits, the characteristics of the required output voltage are obtained with the low and high-frequency operation of the controlled switches^32^. The switching periods of the low-frequency switches depend on the output frequency requirement. The voltage regulation of the output voltage depends on the high-frequency PWM control. The switching periods of the low-frequency control signals are high, and this is not comparable with the unwanted delay generated by the circuit employed for the detection of the zero-crossover point of the input voltage. So, there is no control complication for the synchronization or alignment of the low-frequency control signals with the polarity of the input voltage. However, such synchronization is a big challenge with the generation of high-frequency PWM signals, as their switching periods are low. The minimum number of high-frequency signals in the converters in Refs^21–26^ is two, and they have to be aligned with the polarity variation of the input AC voltage that creates control complexity. The proposed research work introduced a new circuit topology that can perform bipolar voltage gain buck operation with the help of only one high-frequency control signal. The generation of this control signal requires no determination of the polarity of the input voltage. For this purpose, two diodes are connected in series with the source terminals of two high-frequency operating transistors. The drain terminals of these transistors are connected to two different ends of the source voltage. However, the control inputs of these transistors are connected to the same control signals. This means both transistors turn on and off at the same time. The conduction of these transistors depends on the forward biasing of the series of connected diodes, which further depends upon the polarity of the source voltage. The use of diodes determines the polarity sensing of the source, which ensures the independent generation of high-frequency control signals.

The organization of the manuscript includes various sections. Section “Introduction” highlights the introduction that is related to the characteristics of various AC–AC converters. The working principle related to the non-inverted and inverted performance of the proposed circuit is addressed in Sect. “Operation of power circuit and control signals”. This section also includes its mathematical modeling. Section “Comparison-based analysis” compares and analyzes performance indexes of the proposed circuit with similar circuit topologies. The circuit and filter design are detailed in Sect. “Circuit and filter design”. The results obtained from computer simulation and practical setup are presented in Sect. “Results and discussion” to prove the operation of the designed circuit. Section “Conclusion” deals with the conclusion.

## Operation of power circuit and control signals

**Figure 1:**
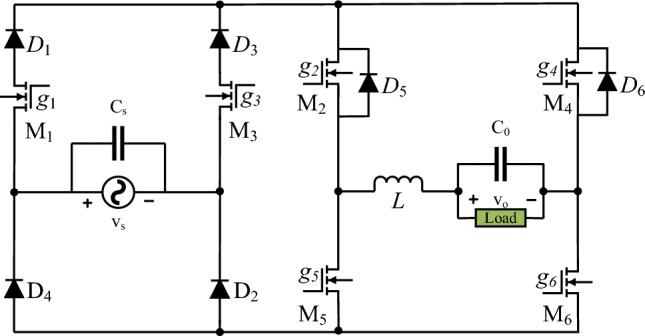
Suggested power circuit.

**Figure 2:**
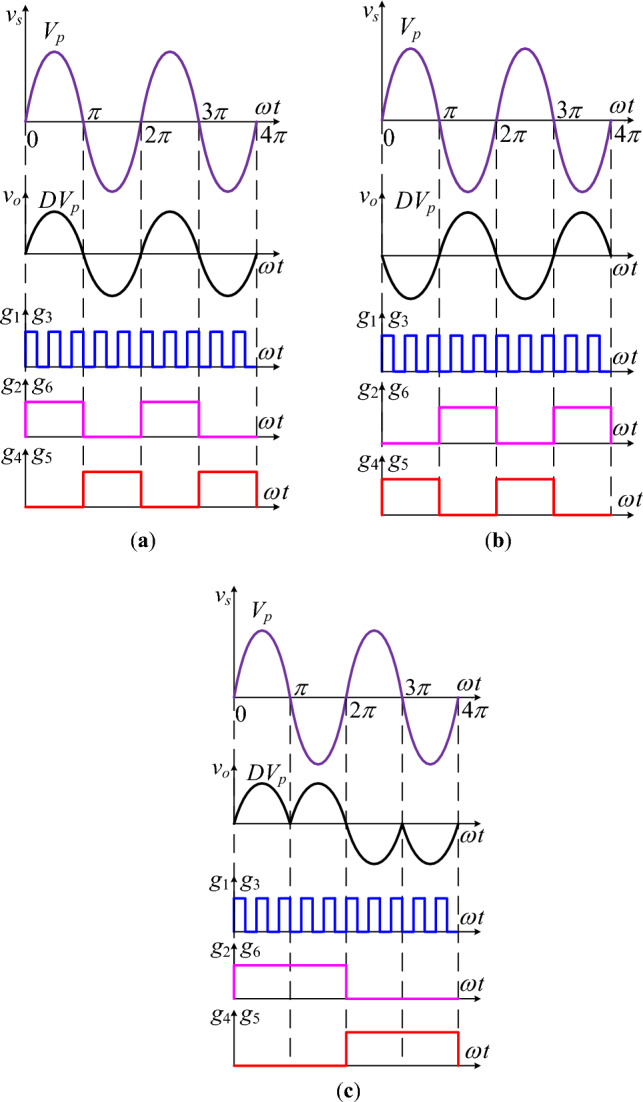
Control signals for; (**a**) noninverting operation; (**b**) inverting operation; (**c**) variable frequency operation.

This section details the operation of the power circuit with the help of control signals. The switching strategy determines the regulation of the output voltage with its noninverting and inverting features. As required, the instantaneous input voltage can be inverted and non-inverted at the output. Figure 1 shows the circuit diagram of the suggested power converter. This circuit is a modified version of the converter developed in^26^. Two transistors *M*_1_ and *M*_2_ are added in series with diodes *D*_1_ and *D*_3_ of the circuit shown in Fig. 2 of Ref^26^. In the same way, two diodes *D*_6_ and *D*_8_ of Fig. 2 are not present in the proposed circuit topology. This means that Fig. 2 of Ref^26^ can be converted to the proposed topology by adding two transistors and removing its two diodes.

The proposed circuit consists of six transistors *M*_1_ to *M*_6_. Some of them can conduct the current only in one direction, and others can conduct it in both directions. Transistors *M*_1_, *M*_3_, *M*_5,_ and *M*_6_ can only conduct the current once they are on. The anode terminals of the diodes *D*_1_ and *D*_3_ are connected with the source terminals of the transistors *M*_1_ and *M*_3,_ respectively. However, the cathode terminals of these diodes are connected to the same point or terminals. As a result, their cathode voltage is equal or the same. The forward or reverse biasing of these two diodes depends on the value of their anode voltages. The voltage at the anode terminals of these two diodes depends on the operation of the transistors *M*_1_ and *M*_3_. The anode terminals of these two diodes are connected to two different terminals of the source voltage once one of their series-connected transistors *M*_1_ and *M*_2_ are on. So, their anode voltages are always unequal or different. This creates a situation for a pair of two diodes that have equal voltage at their cathode terminal but different voltage at their anode terminals. In this scenario, the diode becomes forward-biased, having the highest anode voltage compared to the anode voltage of the other diode. This means that one diode conducts for positive input voltage while the other conducts for negative input voltage. The diodes *D*_2_ and *D*_4_ similarly operate for positive and negative input voltage, respectively. The other transistors *M*_5_ and *M*_6_ conduct current from their drain to source terminals. The transistors *M2* and *M4* have bidirectional current conduction capability due to the parallel connection of diodes *D*_5_ and *D*_6_ across their source and drain terminals. Two capacitors and one inductor are also used. Capacitors are employed to remove the undesirable ripple in the input and output voltage that is generated by the high switching operation of the transistors.

Figure 2a–c demonstrate the plots of required control signals are used to obtain the non-inverted and inverted voltage controllable form of the input voltage at the output. The voltage buck operation is a voltage step-down voltage where the peak or RMS value of the output voltage may be adjusted from a minimum value of the input voltage to close to its maximum value, which is 80 to 90% of the input. This is due to the practical constraints of the operating transistors and gating control circuits. For output voltage regulation, the instantaneous input voltage is converted in the form of high-frequency pulses. This is accomplished with the high-frequency operation of the transistors. The control inputs for the realization of this turn-on and turn-off are shown in Fig. 2 as *g*_1_ and *g*_3_. It can be noted that both signals are the same as they have similar switching characteristics. This control signal is split into two similar isolated signals by employing two gate control circuits as reference terminals of these control signals that are not at the same voltage level. The control of pulse width or duty cycle of these signals regulates the required output voltage. It means that the required voltage gain of the proposed converter is directly related to the pulse width of the high-frequency control signal. The phase or frequency control of the output voltage is completely independent of these two control signals. The operation of the non-PWM control (low frequency) transistor accomplishes the said requirement. For this purpose, two low-frequency signals (*g*_2_, *g*_4_) are generated. The characteristics of these two signals are complementary, and one can be inverted to obtain the other. The turn-on and off intervals of these are equal to half the period of the required output voltage. The role of these low-frequency signals is to turn on the transistors to complete the power transfer path from input terminals to output. The plots of Fig. 2a–c show the control sequence for non-inverted, inverted, and variable frequency operation of the proposed converter. The next subsections detail the operation of the suggested converter.

### Operation for noninverting outputs

**Figure 3:**
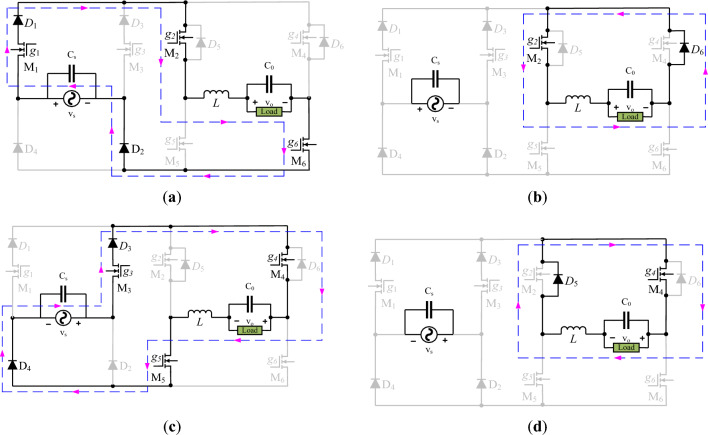
Noninverting circuit operation for; (**a**) and (**b**) positive input; **c**) and **d**) negative input.

This section compressively explains the noninverting operation in which the phase difference between the input and output is zero. It means that the output is positive and negative in voltage-regulated form for positive and negative input voltage, respectively. Such features of the output voltage may be used to increase the terminal voltage of the sensitive load. For this purpose, the decrement in the output voltage is improved with a series addition of converter output voltage. The output terminals of a low-frequency transformer are always connected in series between the sensitive load and source, and its input is connected with the output of the converter. The control in the duty cycle (*d*) of the high-frequency operating transistors adjusts the magnitude of the voltage that needs to be injected into the line. Voltage buck is a voltage step-down operation regulating the output from the maximum voltage level of the source to the lower level. A detailed analysis regarding voltage regulation is divided into two power flow loops during a switching period. The change in switching states draws a boundary between the two operating modes. The polarity of the output voltage is marked, and it is taken as a reference to compare non-inverted and inverted forms of the output voltage. The reference polarity of positive and negative output voltages is marked in Fig. 3a,b, and c,d, respectively, concerning the polarity of the input voltage.

The power flow loops of Fig. 3a,b, and c,d describe the noninverting voltage buck operation for positive and negative half cycles of the input voltage, respectively. Two transistors *M*_2_ and *M*_6_ remain on for the entire positive half-cycle of the input voltage. But, the transistors *M*_1_ and *M*_3_ turn on and off at high switching frequencies, and their role is voltage stabilization. The circuit branch containing a series of connections between transistor *M*_3_ and diode *D*_3_ is non-conducting as the diode *D*_3_ is in reverse bias condition for positive input voltage. The circuit branch containing a series combination of transistor *M*_1_ and diode *D*_1_ can conduct to connect the load with the source via a loop formed with transistors *M*_2_, *M*_6_, inductor (*L*), and load as shown in Fig. 3a. The voltage appearing across the inductor is the difference between the input and output voltage. The turn-on interval is the product of the duty ratio (*d*) and switching period (*T*).

1 $$v_{L} (t = dT) = v_{s} - v_{o}$$

The power transfer from source to load is interrupted by turning off transistor *M*_1_. In this mode, the load consumes the power gained by the inductor. This power transfer loop is shown in Fig. 3b. The diode (*D*_6_) connected across the transistor operates freewheeling, which avoids the current interruption problem. The inductor voltage in this mode may be viewed as.

2 $$v_{L} \left( {t = (1 - d)T} \right) = - v_{o}$$

The polarities of the input and output voltages are changed for negative input voltage. So, the operation of the converter remains non-inverted. In this operation, the role of high-frequency transistor *M*_1_ is interchanged with *M*_3_. The series connected diode *D*_1_ with transistor *M*_1_ ceases its conduction. The switching of transistor *M*_3_ helps to store and release the inductor. These power flow loops are shown in Fig. 3c and d. Voltages across the inductor during these intervals can be viewed by employing KVL loops.

3 $$v_{L} (t = dT) = v_{s} - v_{o}$$

4 $$v_{L} \left( {t = (1 - d)T} \right) = - v_{o}$$

Equations (1), (2), (3), and (4) are analogous to each other as they represent similar circuit behavior that is noninverting. The balancing principle of inductor voltage-second is one of the simple and easy approaches for the determination of voltage gain (*g*_*v*_) in terms of duty ratio control with the given value of the input (*v*_*s*_) and output (*v*_*o*_) voltage.

5 $$\left( {v_{s} - v_{o} } \right)dT = v_{o} \left( {1 - d} \right)T$$

6 $$g_{v} = d = \frac{{v_{o} }}{{v_{s} }}$$

### Operation for inverting outputs

**Figure 4:**
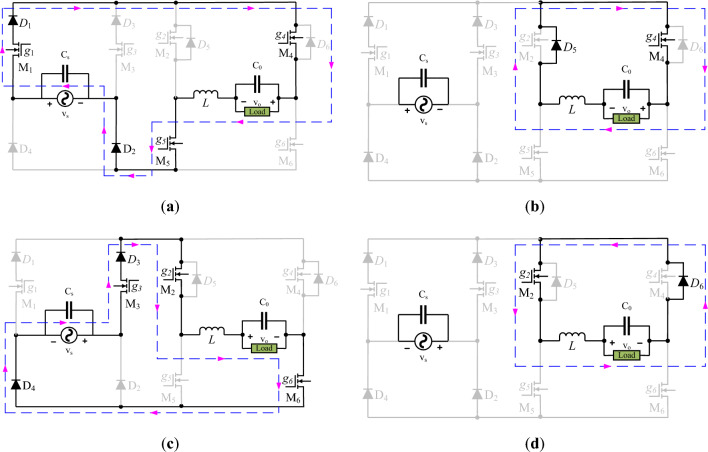
Inverting circuit operation for; (**a**) and (**b**) positive input; (**c**) and (**d**) negative input.

In this operating mode, the load voltage is out of phase concerning the input voltage. It means that the output voltage always exists as an inverted form of the input voltage. The voltage stability of the line voltage can be ensured against over-voltage or voltage swell problems if the line injected voltage has inverting features. The voltage inverting form is realized by inverting the direction of the load current as it was during the noninverting mode. The operation of transistors *M*_1_ and *M*_2_ remains unchanged as their role is only voltage stabilization. The operating role of transistors *M*_2_ and *M*_6_ is swapped with *M*_4_ and *M*_5_ to obtain the inverting output. Figure 4a and b highlight a current conduction path to ensure the voltage inversion of positive input voltage. In this mode, as the polarity of the output voltage is changed, the inductor voltage is the sum of the input and output voltage in the operation of mode no.1.

7 $$v_{L} (t = dT) = v_{s} + v_{o}$$

8 $$v_{L} \left( {t = (1 - d)T} \right) = v_{o}$$

The comparison of these equations with (1) and (2) highlights the change in the polarity sign of output voltage *v*_*o*_. These values of inductor voltages can also be confirmed by applying KVL in the highlighted loops of Fig. 4a and b. A similar mathematical form of inductor voltage is obtained in Eqs. (9) and (10) by considering the current flow loops of Fig. 4c and d.

9 $$v_{L} (t = dT) = v_{s} + v_{o}$$

10 $$v_{L} \left( {t = (1 - d)T} \right) = v_{o}$$

A previous approach may be employed to validate the inverting voltage gain. This includes the current conduction loops for power transfer and the value of the inductor voltage once it stores and releases the power toward the load. These loops are demonstrated in Fig. 4a,b for positive input and in Fig. 4c and d for negative input voltage. The value of the inductor voltage can be simply modified from Eqs. (1) and (2) by just inverting the polarity of the output voltages, and it may be observed from Eqs. (8) and (9).

As can be seen, the inductor volt-second balancing approach for a switching period may lead to obtaining the mathematical form of inverting voltage gain.

11 $$\left( {v_{s} + v_{o} } \right)dT = - v_{o} \left( {1 - d} \right)T$$

12 $$v_{o} \,\,\,\, = - dv_{s}$$

The analytically obtained voltage gain Eqs. (6) and (12) show that output voltage can be realized in noninverting and inverting forms of the input voltage. The controllability of the output voltage can be governed by duty cycle control. This arrangement ensures the voltage regulation in the instantaneous output voltage from maximum input voltage to some lower values.

All the operating modes of the proposed circuit are analyzed deeply in terms of the number of conducting semiconductor devices used in calculating conversion losses and state space modeling. The transfer of input power from the source towards the output is accomplished in one switching interval, i.e., the sum of turn-on and turn-off intervals. These intervals are related to two operating modes during one switching interval. In the first operating interval, input power is stored in the inductor and transferred to the load, and two transistors and two diodes are conducted. But, in the second switching interval, where the stored power of the inductor is released toward the output, only one transistor and diode conduct. In circuit modeling, the internal resistances of transistors, diodes, and inductors are represented as *R*_*T*_, *R*_*D,*_ and *R*_*L,*_ respectively. In the same way, the output resistance and capacitance are represented by *R*_*o*_ and *C*_*o,*_ respectively.

The model based on state space of voltage buck operation is developed and represented in Eqs. (13) and (14) for non-inverted and inverted output voltage, respectively. The development of these analytical equations utilized a similar approach as discussed in Ref^20^. Instantaneous inductor current (*i*_*L*_) and capacitor voltage (*v*_*o*_) are considered state variables, and ‘*d*’ is the duty cycle control used to regulate the output voltage.

13 $$\frac{d}{dt}\left[ \begin{gathered} i_{L} (t) \hfill \\ v_{o} (t) \hfill \\ \end{gathered} \right]\, = \,\left[ \begin{gathered} \frac{{ - \left[ {\left( {R_{T} + R_{D} } \right)(1 + d) + R_{L} } \right]}}{L}\,\,\,\,\,\,\,\,\frac{ - 1}{L} \hfill \\ \frac{1}{{C_{o} }}\,\,\,\,\,\,\,\,\,\,\,\,\,\,\,\,\,\,\,\,\,\,\,\,\,\,\,\,\,\,\,\,\,\,\,\,\,\,\,\,\,\,\,\,\,\,\,\,\,\,\,\,\,\,\,\frac{ - 1}{{R_{o} C_{o} }}\, \hfill \\ \end{gathered} \right]\left[ \begin{gathered} i_{L} (t) \hfill \\ v_{o} (t) \hfill \\ \end{gathered} \right] + \left[ \begin{gathered} \frac{d}{L} \hfill \\ \,0 \hfill \\ \end{gathered} \right]v_{s} (t)$$

14 $$\frac{d}{dt}\left[ \begin{gathered} i_{L} (t) \hfill \\ v_{o} (t) \hfill \\ \end{gathered} \right]\, = \,\left[ \begin{gathered} \frac{{ - \left[ {\left( {R_{T} + R_{D} } \right)(1 + d) + R_{L} } \right]}}{L}\,\,\,\,\,\,\,\,\frac{1}{L} \hfill \\ \frac{1}{{C_{o} }}\,\,\,\,\,\,\,\,\,\,\,\,\,\,\,\,\,\,\,\,\,\,\,\,\,\,\,\,\,\,\,\,\,\,\,\,\,\,\,\,\,\,\,\,\,\,\,\,\,\,\,\,\,\,\,\frac{ - 1}{{R_{o} C_{o} }}\, \hfill \\ \end{gathered} \right]\left[ \begin{gathered} i_{L} (t) \hfill \\ v_{o} (t) \hfill \\ \end{gathered} \right] + \left[ \begin{gathered} \frac{d}{L} \hfill \\ \,0 \hfill \\ \end{gathered} \right]v_{s} (t)$$

## Comparison-based analysis

A simple switching control scheme is one of the main features of the proposed converter. The connection of the semiconductor devices in the developed power converter eliminates the need for alignment of the high-frequency PWM signal with the variation of the input voltage. The required PWM signal can be generated with any simple and low-cost analog or digital signal generator. The pulse width and switching period of this control signal can easily be controlled. The pulse width adjustment helps to set the desired amplitude of the output voltage while the switching period determines the quality of the output voltage. The use of high switching frequency not only improves the power quality but also reduces the size of the filtering components. The single control signal can be transformed into two similar control signals with the help of gate control or drive circuits. The characteristics of the high-frequency PWM control signals remain unaltered for any output frequency requirement.

The data presented in Table 1 compares the attractive features of the developed converters with the existing circuits as reported in Refs^20,26^ in terms of peak voltages, currents, number of devices, components, and conversion losses produced by the power semiconductor devices and passive components. The losses are produced by low- and high-frequency switching devices, which are called conduction and switching losses. These losses are computed and compared during one cycle of the input voltage. The power loss model of the diode includes the forward voltage *V*_*f*_ and forward resistance *R*_*D*_. The conduction losses of the transistor depend on its internal resistance *R*_*T,*_ while the switching losses are a function of switching frequency *f*_*s*_, rise (*t*_*r*_), and fall time (*t*_*f*_). These losses are analyzed by considering the voltage gain of 0.5 for voltage buck operation. In the proposed circuit configuration, during the one complete cycle of the input voltage, two transistors conduct for half a period. The conduction of four high-frequency transistors can be considered the equivalence of two transistors conducting for half a period of the input voltage. This assumption is based on zero rise and fall time. In the same way, the conduction of six high-frequency diodes can also be approximated to three diodes conducting for the half period of the input voltage. The conduction losses of four transistors and three diodes operating in half intervals of the input voltage can be recognized as.

15 $$\begin{gathered} P_{cond} = 3\frac{1}{2\pi }\int\limits_{0}^{\pi } {V_{f} } I_{L(P)} \sin \left( {\omega t} \right)d\left( {\omega t} \right) + 3\frac{1}{2\pi }\int\limits_{0}^{\pi } {I_{L(P)}^{2} } \sin^{2} \left( {\omega t} \right)R_{D} d\left( {\omega t} \right) \hfill \\ \,\,\,\,\,\,\,\,\,\,\,\,\,\,\,\, + 3\frac{1}{2\pi }\int\limits_{0}^{\pi } {I_{L(P)}^{2} } \sin^{2} \left( {\omega t} \right)R_{T} d\left( {\omega t} \right) \hfill \\ \,\,\,\,\,\,\,\,\,\,\, = \frac{{3V_{f} I_{L(P)} }}{\pi } + \frac{{3I_{L(P)}^{2} R_{D} }}{4} + I_{L(P)}^{2} R_{T} \hfill \\ \end{gathered}$$

**Table 1:** Performance analysis and comparison.

Parameters	Proposed circuit	Converter of ^20^	Converter in ^26^	Converter in ^28^
Peak currents value	$I_{L(P)}$	$I_{L(P)}$	$I_{L(P)}$	$I_{L(P)}$
Current interruption of the inductor	No	No	No	Yes
Bipolar voltage gain control	Yes	Yes	Yes	No
Switching current (*I*_*SW*_)	*I*_*o*_	*I*_*o*_	*I*_*o*_	*1.5 I*_*o*_
Switching voltage (*V*_*SW*_)	$V_{{SW(D_{1} + M_{1} ,\,\,D_{3} + M_{3} )}} = 2V_{o}$ $V_{{SW(D_{2,4,5,6} \,{\text{and}}\,M_{2,4,5,6} ,)}} = 2V_{o}$	$V_{{SW({\text{all}}\,{\text{transistors}}\,\,{\text{and}}\,\,{\text{diodes}})}} = 2V_{o}$	$V_{{SW({\text{Diodes + Transistors}})}} = 2V_{o}$	$V_{SW} = 3V_{o}$
Maximum conducting transistors for one switching period	4	4	3	3
Maximum conducting diodes in one switching period	3	4	3	3
Synchronization of PWM signals with input voltage	No	Yes	Yes	Yes
Number of capacitors	2	2	2	2
Number of inductors	1	1	1	2
Conduction-losses	$$\begin{gathered} \frac{{3\sqrt 2 V_{f} I_{o} }}{\pi } + \frac{{3I_{o}^{2} R_{D} }}{2} \hfill \\ + 2I_{o}^{2} R_{T} \hfill \\ \end{gathered}$$	$$\begin{gathered} \frac{{3\sqrt 2 V_{f} I_{o} }}{\pi } + \hfill \\ \frac{{3I_{o}^{2} }}{2}\left( {R_{D} + R_{T} } \right) \hfill \\ \end{gathered}$$	$$\begin{gathered} \frac{{6\sqrt 2 V_{f} I_{o} }}{\pi } + 3I_{o}^{2} R_{D} \hfill \\ + 2I_{o}^{2} R_{T} \hfill \\ \end{gathered}$$	$$\begin{gathered} \frac{{3\sqrt 2 V_{f} I_{o} }}{\pi } \hfill \\ + \frac{{9I_{o}^{2} \left( {R_{D} + R_{T} } \right)}}{4} \hfill \\ \end{gathered}$$
Switching-losses	$P_{sw} = \frac{4}{3}V_{o} I_{o} f_{s} \left( {t_{r} + t_{f} } \right)$	$P_{sw} = \frac{2}{3}V_{o} I_{o} f_{s} \left( {t_{r} + t_{f} } \right)$	$P_{sw} = \frac{4}{3}V_{o} I_{o} f_{s} \left( {t_{r} + t_{f} } \right)$	$3V_{o} I_{o} f_{s} \left( {t_{r} + t_{f} } \right)$

The peak inductor current in voltage buck operation is to load current, which means *I*_*L*(*P*)_ = *I*_*o*(*P*),_ and RMS of the output current is related to its peak current as

16 $$I_{o(P)} = \sqrt 2 I_{o}$$

The total conduction losses with respect to the RMS value of the output current may be recognized as.

17 $$P_{cond} = \frac{{3\sqrt 2 V_{f} I_{o} }}{\pi } + \frac{{3I_{o}^{2} R_{D} }}{2} + 2I_{o}^{2} R_{T}$$

The switching losses of the diodes depend on their reverse recovery behavior, and they have abrupt recovery characteristics when employed as high-switching diodes. They have very low reverse recovery time and charge so their switching losses are not comparable to the switching losses of the transistors. The switching losses of the two transistors operating at line frequency can also be neglected. Therefore, switching losses of the remaining four high switching speed transistors can be viewed by approximating the switching voltage to RMS input voltage and switching current to RMS inductor current that is equal to RMS output current in voltage buck operation.

18 $$P_{sw} = \frac{4}{3}V_{o} I_{o} f_{s} \left( {t_{r} + t_{f} } \right)$$

In the proposed converter, the filtering inductor ‘*L*’ is only one passive component in which continuous current is flowing in a voltage buck converter; its RMS current can be approximated to output RMS current by ignoring the peak-to-peak inductor ripple current. This is because of the selection of high switching frequency. The power losses of the filtering inductor can be calculated as.

19 $$P_{ind} = I_{o}^{2} R_{ind}$$

Output power depends on the output RMS voltage, RMS current, and load power factor.

20 $$P_{0} = V_{o} I_{o} \cos \left( {\theta_{o} } \right)$$

It can be viewed that the switching losses depend on the switching frequency (lies in kHz) as well as rise and fall times that have nanosecond values in high switching transistors. These losses in modern transistors cannot contribute significantly. In a similar way, the power losses produced by the passive components can also be ignored as they have low internal resistances. The overall conversion losses of the converter are contributed by the conduction losses. Input power is the sum of output power and losses. The overall efficiency of the system by ignoring the switching losses and losses of the filtering component is computed as.

21 $$\eta = \frac{{V_{o} I_{o} \cos \left( {\theta_{o} } \right)}}{{V_{o} I_{o} \cos \left( {\theta_{o} } \right) + P_{cond} }}$$

**Figure 5:**
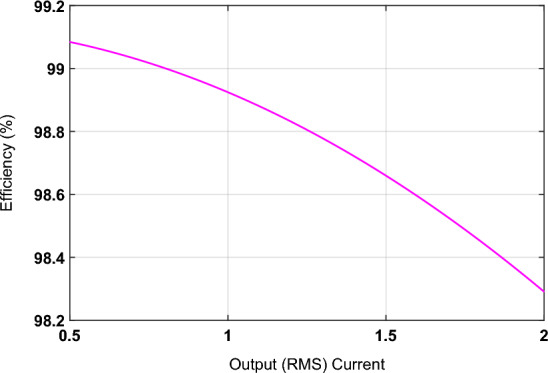
Efficiency versus load current.

Figure 5 shows the variation in the efficiency by varying load current with unity power factor load and the value of duty cycle 0.5. The value of the diode forward voltage and transistor’s resistance is taken as 1.5 V and 0.25 Ω. The value of the diode forward resistance can also be ignored.

22 $$\eta = \frac{{V_{o} I_{o} \cos \left( {\theta_{o} } \right)}}{{V_{o} I_{o} \cos \left( {\theta_{o} } \right) + 1.35V_{f} I_{o} + 2I_{o}^{2} R_{T} }}$$

It is observed from Eq. (22) that the efficiency of the proposed controlled converter with constant output voltage decreases with an increase in load current. The dropping characteristics of the plot in Fig. 5 also confirm this behavior.

A similar procedure can also be adopted for the converter in Ref^28^ to compute the conduction losses and switching losses for a voltage gain of 0.5 in Eqs. (23), (24).

23 $$P_{cond} = \frac{{3\sqrt 2 V_{f} I_{o} }}{\pi } + \frac{{9I_{o}^{2} \left( {R_{D} + R_{T} } \right)}}{4}$$

24 $$P_{sw} = 3V_{o} I_{o} f_{s} \left( {t_{r} + t_{f} } \right)$$

The peak-to-peak inductor ripple current for the proposed converter and converter in Ref^28^ is recognized as

25 $$\Delta I_{L(proposed)} = \frac{{V_{o} }}{{f_{s} L}}$$

26 $$\Delta I_{L[28]} = \frac{{3V_{o} }}{{2f_{s} L}}$$

The conducted research streamlines the limitations of similar existing topologies and enhances its potency for application in various industrial drives for voltage as well as frequency regulation or control. Table 1 demonstrates the comparison of the proposed circuit with some similar circuits in terms of performance parameters.

The conversion losses of the suggested topology are improved as in Ref^20^ and almost the same as in Refs^26,28^. However, the voltage regulation approach in the proposed circuit is much simpler and easier compared to the circuit reported in the literature. The high-frequency PWM control signals are applied and generated as in DC converters as they need not align with the polarity variation of the AC source voltage. It is one of the attractive features for the analysis of the feedback control. Also, the configuration of the converter is developed in such a way that it always avoids the current interruption problem of the filtering inductor. That eliminates the risk of generation of high voltage surge and saves the semiconductor switching devices from failure.

## Circuit and filter design

The design procedure of the proposed circuit is detailed below by following or adapting a similar approach in Ref^26^. The design of the power electronics circuit always includes the determination of the voltage and current rating. That is the determination of their peak, RMS, and average values. The other part of the design is to evaluate the value of the passive elements that are determined for their ripple contents. The role of the output capacitor (*C*_*o*_) is to deal with the load ripple voltage at a given switching frequency for power quality improvement. This capacitor is also responsible for supplying the reactive power demand in case of the inductive load. The capacitor employed across the input source voltage is also responsible for the elimination of the grid side ripple. It also maintains the continuity of the input current. In this way, it performs the filtering operation to improve the power quality indexes of the source current. The inductor connected in series with the parallel combination of load and capacitor is an energy-storing element as it energizes and reenergizes in PWM on and off intervals, respectively. In the PWM off period, it maintains the continuity of the load current by releasing its stored energy. At the same time, the capacitor connected across the load becomes the source, and it supplies power to the load.

The effect of the peak-to-peak inductor current is represented in the form of the inductor ripple factor (*k*_*ind*_). In the same way, the presence of the output ripple is measured as capacitor factor (*k*_*cap*_). Their values are taken as 20% and 5% to maintain the power quality at an acceptable index level. The value of switching frequency, duty ratio, source RMS voltage, inductor RMS current, and input RMS current for the design of the passive filtering components are selected as 30 kHz, 0.5, 230 V, 6 A, and 3 A, respectively. The value of the filter inductor is calculated by assuming these values as

27 $$L = \,\frac{{V_{s} k(1 - k)}}{{k_{ir} I_{{{\text{in}} d}} f_{s} }}\, = 1.6\,{\text{mH}}$$

The following equation links the output ripples with the output value. This relation can be utilized to find the capacitor value to maintain its ripple within an acceptable level by assuming the stated values.

28 $$C_{o} = \,\frac{{kI_{o} }}{{k_{vr} V_{o} f_{s} }}\, = 17.40\,{\mu F}$$

The value of the input capacitor may also be realized by assuming the same procedure and values.

29 $$C_{s} = \,\frac{{I_{s} (1 - k)}}{{k_{vr} V_{s} f_{s} }}\, = 4.34\,{\mu F}$$

These calculated values approximate to 1.5 mH, 4.7 µF, and 20 µF for inductor filter, source, and output capacitor filters, respectively.

## Results and discussion

Computer simulation is a powerful tool to endorse the validation aspects of the circuit under test. Then, these results may be verified using the results obtained from the mathematical environment and practical test bench. This section includes the results that explore the non-inverted and inverted features of the converter under consideration.

### Simulation results

Computer simulation is one of the simple and accurate approaches to simulate the converter that is under investigation in terms of its performance. This platform facilitates the circuit designer in modeling the switching converter according to the data sheet of the switching devices and constraints of passive components. The circuit can be easily remodeled and analyzed in a short time interval. In this practice, the value of the input voltage, load resistance, forward voltage drops, and internal resistances of the semiconductor devices are set according to the requirement of a practical test circuit and the catalog of the devices used. The value of the voltage gain in all test results is taken as 0.5. The target of this voltage gain is to convert 330 peak input voltage to 165 peak output voltage, which may be a non-inverted and inverted form of the input voltage. In this power conversion process, a load value of 150 Ω is used to keep current in the operating devices within a safe limit. With this arrangement, the peak load and input currents are almost near 1 A and 0.5 A, respectively.

**Figure 6:**
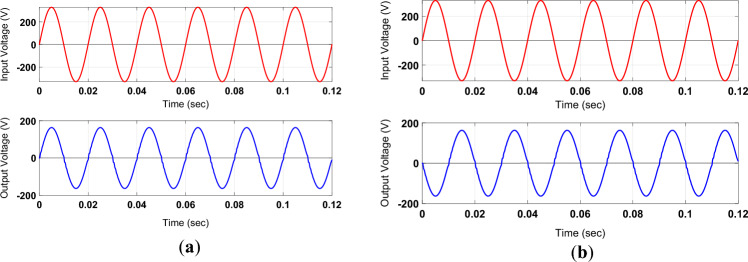
Simulation-based outputs for voltage gain of 0.5 in (**a**) non-inverted form; (**b**) inverted form.

The results of Fig. 6a and b endorse the non-inverted and inverted conversion of the input voltage at the output with a voltage gain of 0.5.

**Figure 7:**
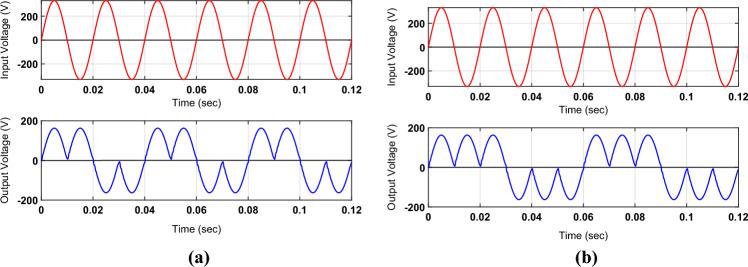
Simulation-based outputs for variable frequency operation. (**a**) one-half of input frequency; (**b**) one-third of input frequency.

The results shown by the plots of Fig. 7a and b integrate the noninverting and inverted characteristics of the proposed converter to govern output frequency at a discrete level. The output frequencies of the outputs shown in Fig. 7a and b are one-half and one-third of the input frequency, respectively. These waveforms are integrated by taking the non-inverted and inverted forms of positive and negative input voltage. The use of inductors and capacitors as filtering and energy-storing elements maintains the ripple in all output voltage waveforms within the acceptable level.

**Figure 8:**
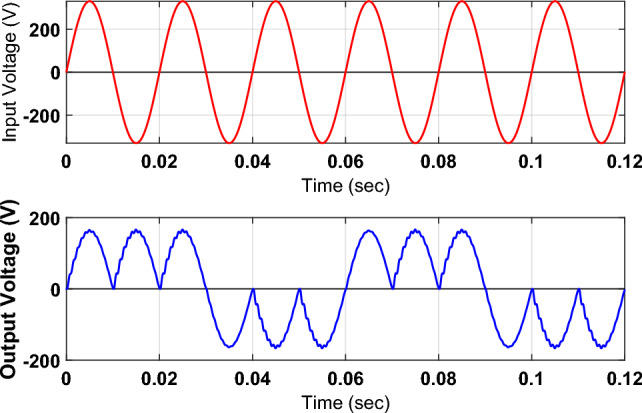
Simulation-based outputs voltage waveform for inductive load.

The proposed topology may also be effectively employed for an inductive load like an induction motor. The capacitor connected across the load not only suppresses the ripple component in the load voltage but also compensates the load reactive power demand. Without an output capacitor, an inductive load demands active power as well as reactive power that may flow back and forth between the load and the source. The value of the supplied current increases due to the flow of active and reactive power from the source to the load, and the power factor lags. However, the connection of a capacitor across the load supplies reactive power demand reduces the flow of current from source to load and improves the power factor. Figure 8 shows the simulated output voltage for an inductive load having a resistance of 30 Ω and inductance of 10 mH.

The analysis of these output waveforms endorses the validity of the required outcomes of the suggested topology. It includes the adjustment of the output voltage through PWM control and frequency regulation by setting the periods of the low-frequency control signals.

### Practical results

**Figure 9:**
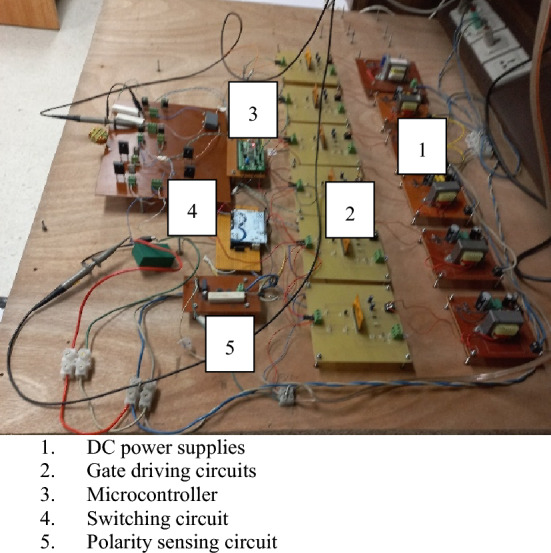
Picture of the test bench.

This section describes the practical test circuit and the results of the conversion of the non-inverted and inverted form of input voltage with the required voltage gain. In a high-switching converter, the presence of the filtering inductor is mandatory, and its role is to store and release the energy during turn-on and turn-off intervals of high-frequency PWM control signal. The value of this inductor helps to lower the ripple in the output and input current, which helps to improve their power quality. In this regard, two inductors of 0.5 mH and 1.5 mH are employed for the input and output side, respectively. In the same way, the suppression of ripple in the voltage needs the use of capacitors with values of 20 µF and 4.7 µF capacitors selected for the output and input side, respectively. The value of these filtering components (inductors and capacitors) is designed according to the requirement. For semiconductor devices, six high power rating fast recovery diodes (RHRG3060) and six fast switching transistors (IRF840) are used to implement the practical test circuit. The gate drive circuits are an integral part of the high-switching converters. It is not possible for any digital controller, either a micro or Arduino controller, to directly govern the operating states of switching transistors as the maximum voltage level of these controllers is limited to a 5-V level. A hybrid chip (EXB840) is used as a driving circuit for one switching transistor. The magnitude of the output pulse of this chip is set according to the switching transistor. For transistor IRF 840, it may be up 20 V. Such control circuits should produce isolated output as the reference terminal of all (six) switching transistors may not have the same voltage level. For this purpose, an isolated DC power supply is required to operate an individual gate control circuit. The picture of the test bench is shown in Fig. 9.

**Figure 10:**
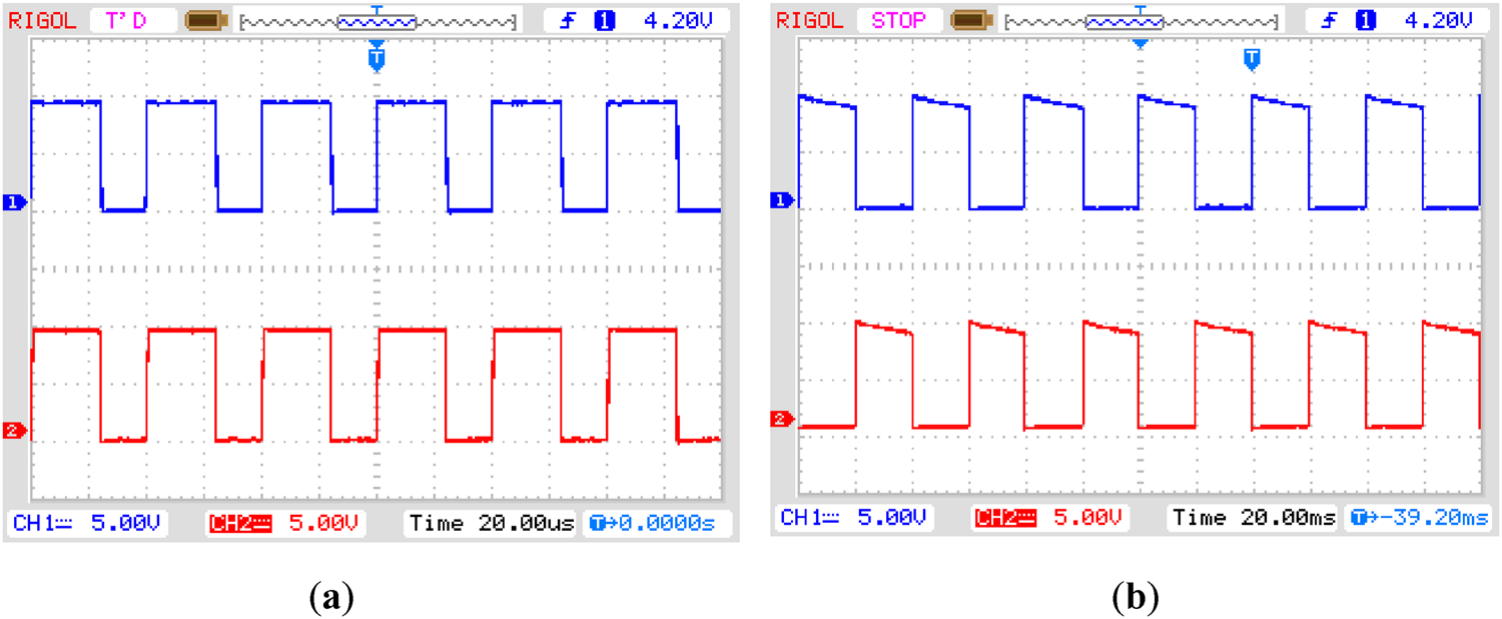
Practically obtained control signals for operation of (**a**) high-frequency transistors (**b**) low-frequency transistors.

The practically generated gate control signals produced by gate driving circuits are shown in Fig. 10. They are obtained by employing a Rigol oscilloscope. The maximum value of these control signals is almost 10 V. This value can be adjusted by setting the turn ratio of the transformer employed in an isolated DC supply. The control outputs shown in Fig. 10a are high-frequency PWM signals that are used to set the magnitude or RMS value of the output voltage to any desired value ranging from the minimum value of the source voltage to its maximum value. For this purpose, the pulse width of these signals is adjusted only without changing its period. The characteristics of these two signals are similar, as one control output of the microcontroller is split into two control signals by employing two separate gate control circuits. Two transistors connected in two different branches of the circuit will employ these control signals. The control signals shown in Fig. 10b are low-frequency signals connected to two pairs of transistors. Their role is only to govern the output voltage frequency in discrete values. This is obtained by controlling the period of these waveforms. These control signals are also employed to produce the non-inverted or inverted form of output. In summary, it may be concluded that in the proposed circuit topology, the voltage and frequency control of the output voltage are independent, and their values are governed separately.

**Figure 11:**
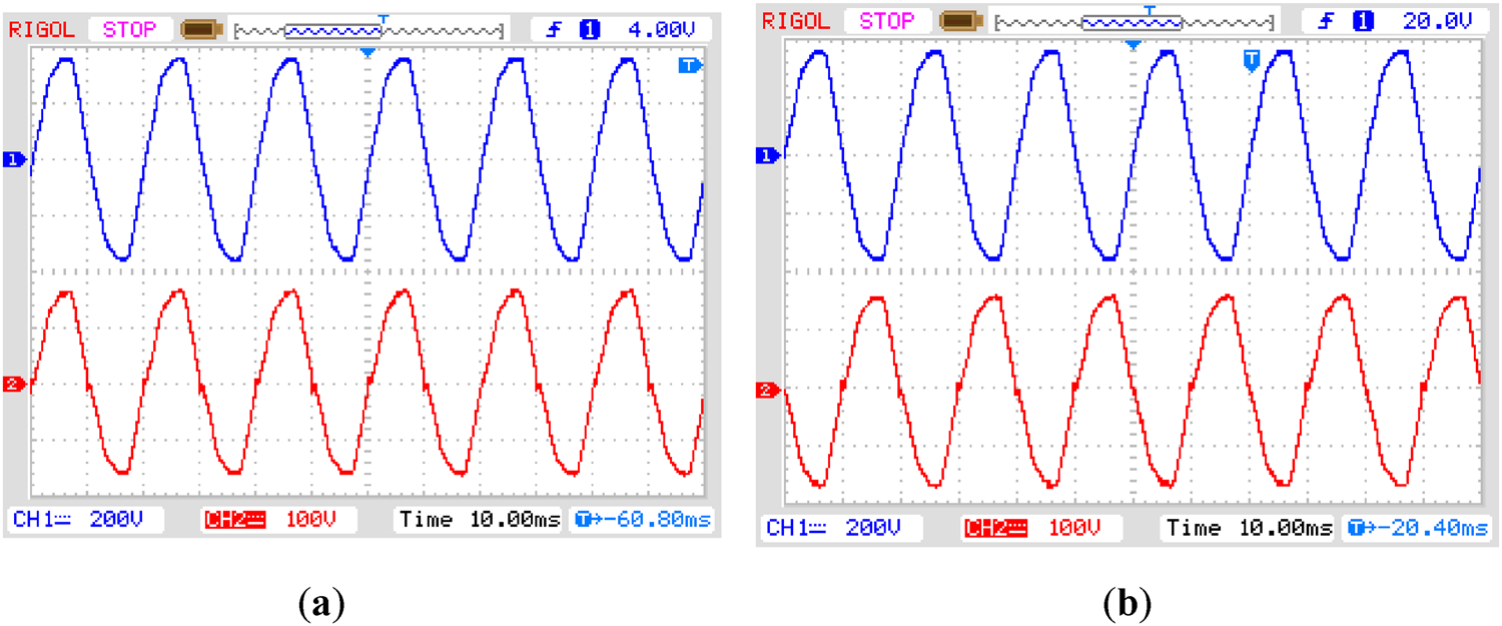
Practically obtained results of output voltage (red) concerning the input voltage (blue) in (**a**) non-inverted form; (**b**) inverted form.

The plots of Fig. 11a and b represent the non-inverted and inverted forms of the input voltage with the value of the voltage gain of 0.5. It means that the peak value of the input voltage is two times that of the output voltage. In this case, the peak values of the input and output voltages are 330 V and 165 V, respectively. There is no significant observation in the change of power quality of these two waveforms except the zero cross-over point of the output voltage. This is because of the series connection of the output filter inductor with the load, as the inductor current has to change its direction once the polarity of the output voltage is changed. There is no significant interruption or change in the inductor current as the magnitude of the output voltage during these intervals is almost zero.

**Figure 12:**
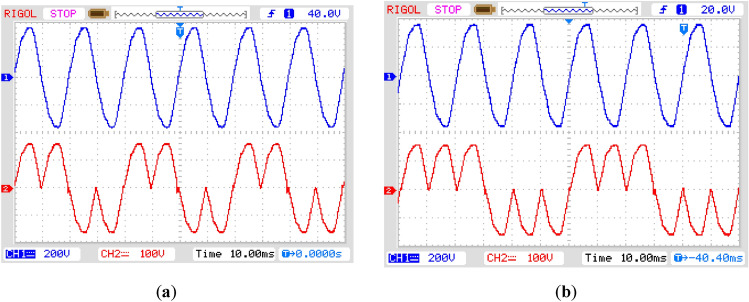
Practically obtained results for output frequency (red) concerning input voltage (blue) (**a**) one-half of input; (**b**) one-third of input.

The non-inverted and inverted form of input voltage with its polarity (positive or negative) is utilized for some positive or negative half cycles to obtain the variation of load frequency in discrete steps. The plot of Fig. 12a and b is recognized by arranging consecutive positive and negative half cycles of two and three, respectively. This organization of the half cycles sets the period of the output voltage two and three times that of the period of the source voltage. This results in the output frequency setting of one-half and one-third of the input frequency. By following a similar method, other discrete values of the output frequency may also be produced.

The achievement of practical and simulation-based results endorses the proposed circuit’s effective validity and control topology.

## Conclusion

This research presents a simplified circuit and control topology related to a single-phase direct AC–AC converter. The developed topology can produce the voltage-regulated form of input voltage with non-inverted and inverted characteristics. Such output features are obtained with the low and high-frequency operation of the fully controlled operating transistors. The operation of the low-frequency transistor is to govern the output frequency requirements. For this purpose, the periods of the low-frequency controlled signals are controlled as per requirement. The value of these periods for the low output frequency application is low source voltages. So, there is no control complication in aligning these gating signals with the input voltage. On the other hand, the output voltage’s magnitude adjustment requires the switching transistors’ high-frequency operation. Reducing the size of filtering components and the ripple contents in the inductor ripple current and capacitor ripple voltage is achieved through high switching frequency. The low ripple in the current and voltage indicates improved power quality. However, aligning high-frequency gating signals with the polarity of the source voltage is a challenging issue as the gating signal period is much lower than that of the period of the source voltage. The generated high-frequency gating signals are free from the polarity change of the input voltage. For this purpose, two fast recovery diodes are connected in series with high-frequency operating transistors. The input terminals of these transistors are connected to the single control signal. This arrangement enables the connection of anodes of two series connected diodes with the two (positive and negative) terminals of the source voltage once the high-frequency operating transistors are on. The polarity of the anode voltage of these series-connected diodes changes, and their forward and reverse biasing states change once the polarity of the source voltage changes. Therefore, one series connected branch conducts during the positive half period while the other during the negative half cycle of the source voltage. The forward and reverse biasing of the diodes with control input eliminates the requirement of polarity sensing in generating high-frequency gating signals, thus solving the control complication issue. The operation of all modes of operation, along with voltage gain, is comprehensively explored. Both the simulation and experimental evidence support the functioning of the suggested circuit.

## Data Availability

The datasets used and/or analyzed during the current study are available from the corresponding author upon reasonable request.

## References

[1] Petrauskas G, Svinkunas G (2022). Application of single-phase supply AC-DC-AC VFD for power factor improvement in LED lighting devices loaded power distribution lines. Appl. Sci..

[2] Shahzad D, Pervaiz S, Zaffar NA, Afridi KK (2021). GaN-based high-power-density AC–DC–AC converter for single-phase transformerless online uninterruptible power supply. IEEE Trans. Power Electron..

[3] da Costa AEL, Jacobina CB, Rocha N, da Silva ERC, de Moura Lacerda Filho AV (2020). A single-phase AC–DC–AC unidirectional three-leg converter. IEEE Trans. Ind. Electron..

[4] Lucena AE, da Costa N, de Moraes S, Marinus L, Jacobina CB, Rocha N (2023). A single-to-three-phase 12-switch AC–DC–AC converter. IEEE Trans. Ind. Electron..

[5] Liu F, Ruan X, Huang X, Qiu Y, Jiang Y (2022). Control scheme for reducing second harmonic current in AC–DC–AC converter system. IEEE Trans. Power Electron..

[6] de Lacerda RP, Jacobina CB, Fabricio ELL, Felinto AS, Cardoso JT (2023). Single-phase AC-DC-AC multilevel five-leg converter with high-frequency link. IEEE Trans. Ind. Appl..

[7] Cardoso JT, Jacobina CB, Rodrigues PLS, Lima AMN (2023). Single-phase AC-DC-AC multilevel five-leg converter based on a high-frequency transformer. IEEE Trans. Ind. Appl..

[8] Ashraf N, Abbas G, Abbassi R, Jerbi H (2021). Power quality analysis of the output voltage of AC voltage and frequency controllers realized with various voltage control techniques. Appl. Sci..

[9] Peng FZ, Chen L, Zhang F (2003). Simple topologies of PWM AC-AC converters. IEEE Power Electron. Lett..

[10] Fang XP, Qian ZM, Peng FZ (2005). Single-phase Z-source PWM AC-AC converters. IEEE Power Electron. Lett..

[11] Aleem Z, Yang H-K, Ahmed HF, Winberg SL, Park J-W (2021). A class of single-phase Z-source AC–AC converters with magnetic coupling and safe-commutation strategy. IEEE Trans. Industr. Electron..

[12] Nguyen M-K, Lim Y-C, Kim Y-J (2012). A modified single-phase quasi-Z-source AC–AC converter. IEEE Trans. Power Electron..

[13] He L, Nai J, Zhang J (2018). Single-phase safe-commutation trans-Z-source AC–AC converter with continuous input current. IEEE Trans. Industr. Electron..

[14] Ellabban O, Abu-Rub H, Bayhan S (2016). Z-source matrix converter: an overview. IEEE Trans. Power Electron..

[15] Ashraf N, Abbas G, Raza A, Ullah N, Mohammad A, Farrag ME (2022). A single-phase compact-sized matrix converter with symmetrical bipolar buck and boost output voltage control. Energies.

[16] Kaniewski J, Szczesniak P, Jarnut M, Benysek G (2015). Hybrid voltage sag\/swell compensators: a review of hybrid AC\/AC converters. IEEE Ind. Electron. Mag..

[17] Li P, Hu Y (2017). Unified noninverting and inverting PWM AC–AC converter with versatile modes of operation. IEEE Trans. Industr. Electron..

[18] Subramanian S, Mishra MK (2010). Interphase AC–AC topology for voltage sag supporter. IEEE Trans. Power Electron..

[19] Khan UA, Khan AA, Cha H, Kim H, Kim J, Baek J (2018). Dual-buck AC–AC converter with inverting and noninverting operations. IEEE Trans. Power Electron..

[20] Ashraf N, Izhar T, Abbas G, Awan AB, Farooq U, Balas VE (2020). A new single-phase AC voltage converter with voltage buck characteristics for grid voltage compensation. IEEE Access.

[21] Abdoli I, Mosallanejad A (2022). A highly efficient isolated single-phase variable frequency AC–AC converter with flexible buck-boost factor, inherent safe commutation, and continuous current. IET Power Electron..

[22] Sharifi S, Monfared M, Nikbahar A (2021). Highly efficient single-phase direct AC-to-AC converter with reduced semiconductor count. IEEE Trans. Industr. Electron..

[23] Ahmed HF, Cha H, Khan AA, Kim J, Cho J (2017). A single-phase buck-boost matrix converter with only six switches and without commutation problem. IEEE Trans. Power Electron..

[24] Ashraf N, Abbas G, Khan I, Raza A, Ullah N (2021). A transformer-less multiconverter having output voltage and frequency regulation characteristics, employed with simple switching algorithms. Appl. Sci..

[25] Ahmed HF, El Moursi MS, Zahawi B, Hosani KA (2021). High-efficiency single-phase matrix converter with diverse symmetric bipolar buck and boost operations. IEEE Trans. Power Electron..

[26] Ashraf N, Abbas G, Ullah N, Al-Ahmadi AA, Mohammad A, Farooq U (2022). A simple circuit and control topology to produce bipolar non-inverted and inverted voltage step-down features. Appl Sci.

[27] Nandakumar K, Mohan V, Alsaif F, Senthilkumar S (2024). Design and analysis of solitary AC–AC converter using reduced components for efficient power generation system. Sci. Rep..

[28] Adel F, Lashine AE, El-Sabbe AE, Osheba DS (2023). A single-phase direct buck-boost AC–AC converter with minimum number of components. Sci Rep.

[29] Osheba DSM, Ahmed SM, Lashine AE (2023). Analysis and experimental validation of single-phase cascaded boost AC–AC converter with high voltage gain. Sustainability.

[30] Chakroun J, Hamam H (2018). General study for energy recovery from used batteries using fuzzy logic and PI controllers. Int. J. Intell. Eng. Inf..

[31] Sadiq U (2024). A machine learning-based solution for monitoring of converters in smart grid application. Int. J. Adv. Comput. Sci. Appl..

[32] Ahmed HF, Alzaabi O, El Moursi MS, Al Hosani K (2023). Highly efficient dual-buck structured buck-boost AC–AC converter with versatile identical inverting/noninverting operations. IEEE Trans. Ind. Inf..

